# Microglial inclusions and neurofilament light chain release follow neuronal α-synuclein lesions in long-term brain slice cultures

**DOI:** 10.1186/s13024-021-00471-2

**Published:** 2021-08-11

**Authors:** Melanie Barth, Mehtap Bacioglu, Niklas Schwarz, Renata Novotny, Janine Brandes, Marc Welzer, Sonia Mazzitelli, Lisa M. Häsler, Manuel Schweighauser, Thomas V. Wuttke, Deborah Kronenberg-Versteeg, Karina Fog, Malene Ambjørn, Ania Alik, Ronald Melki, Philipp J. Kahle, Derya R. Shimshek, Henner Koch, Mathias Jucker, Gaye Tanriöver

**Affiliations:** 1 DZNE, German Center for Neurodegenerative Diseases, 72076 Tuebingen, Germany; 2 Department of Cellular Neurology, Hertie Institute for Clinical Brain Research, University of Tuebingen, 72076 Tuebingen, Germany; 3 Graduate Training Center of Neuroscience, University of Tuebingen, 72076 Tuebingen, Germany; 4 Department of Neurology and Epileptology, Hertie Institute for Clinical Brain Research, University of Tuebingen, 72076 Tuebingen, Germany; 5 Department of Neurosurgery, University of Tuebingen, 72076 Tuebingen, Germany; 6 Division of Neuroscience, H. Lundbeck A/S, 2500 Valby, Denmark; 7 MIRCen, CEA and Laboratory of Neurodegenerative Diseases, CNRS, Institut François Jacob, 92265 Fontenay-aux-Roses, France; 8 Laboratory of Functional Neurogenetics, Department of Neurodegeneration, Hertie-Institute for Clinical Brain Research, University of Tuebingen, 72076 Tuebingen, Germany; 9 Neuroscience Research, Novartis Institutes for BioMedical Research, CH-4056 Basel, Switzerland; 10 Department of Epileptology, Neurology, RWTH Aachen University, Aachen, Germany

**Keywords:** Alpha-synuclein, Microglia, Neurofilament light chain, Slice culture

## Abstract

**Background:**

Proteopathic brain lesions are a hallmark of many age-related neurodegenerative diseases including synucleinopathies and develop at least a decade before the onset of clinical symptoms. Thus, understanding of the initiation and propagation of such lesions is key for developing therapeutics to delay or halt disease progression.

**Methods:**

Alpha-synuclein (αS) inclusions were induced in long-term murine and human slice cultures by seeded aggregation. An αS seed-recognizing human antibody was tested for blocking seeding and/or spreading of the αS lesions. Release of neurofilament light chain (NfL) into the culture medium was assessed.

**Results:**

To study initial stages of α-synucleinopathies, we induced αS inclusions in murine hippocampal slice cultures by seeded aggregation. Induction of αS inclusions in neurons was apparent as early as 1week post-seeding, followed by the occurrence of microglial inclusions in vicinity of the neuronal lesions at 2–3 weeks. The amount of αS inclusions was dependent on the type of αS seed and on the culture’s genetic background (wildtype vs A53T-αS genotype). Formation of αS inclusions could be monitored by neurofilament light chain protein release into the culture medium, a fluid biomarker of neurodegeneration commonly used in clinical settings. Local microinjection of αS seeds resulted in spreading of αS inclusions to neuronally connected hippocampal subregions, and seeding and spreading could be inhibited by an αS seed-recognizing human antibody. We then applied parameters of the murine cultures to surgical resection-derived adult human long-term neocortical slice cultures from 22 to 61-year-old donors. Similarly, in these human slice cultures, proof-of-principle induction of αS lesions was achieved at 1week post-seeding in combination with viral A53T-αS expressions.

**Conclusion:**

The successful translation of these brain cultures from mouse to human with the first reported induction of human αS lesions in a true adult human brain environment underlines the potential of this model to study proteopathic lesions in intact mouse and now even aged human brain environments.

**Supplementary Information:**

The online version contains supplementary material available at 10.1186/s13024-021-00471-2.

## Background

Synucleinopathy is a collective term for neurodegenerative diseases such as Parkinson’s disease (PD), dementia with Lewy bodies (DLB), and multiple system atrophy (MSA). Initial evidence for a cell-non-autonomous mechanism underlying synucleinopathies came from the discovery of Lewy pathology in grafted cells in post mortem brains of PD patients [1, 2]. Therefore, prion-like transmission of αS lesions was proposed and the concept of seeded aggregation – a nucleation-dependent process similar to the one described for prions and amyloid-beta (Aβ) – was suggested [3, 4]. This concept gained further support by in vitro findings showing that the exogenous application of αS fibrils induced Lewy body-like pathology in cultured neurons [5–8]. Similarly, induction and spreading of αS lesions was reported in mice using recombinant αS preformed fibrils (αS pff) or brain homogenates containing aggregated αS [9–15].

Induction and propagation of proteopathic lesions are largely dependent on the interaction between the seed and the host environment [16]. Spreading of αS lesions occurs along neural pathways and cell-to-cell transfer is cell-type dependent and promoted by neural activity [11, 17–19]. Thus, seed propagation is best studied in a living environment that closely mimics the adult or aged brain. While mice have been instrumental in the past to study such prion-like propagation of pathogenic seeds [16], mouse models are time-consuming and costly, and experimental manipulations to mechanistically understand propagation of seeds are challenging.

Studying propagation of pathogenic seeds in humans is even more intricate. Human stem cell-derived organoids and 3D-culture systems have been developed to mimic the human brain environment [20–22], however, they remain controversial regarding their purity, maturity, and cell subtype-identity. Grafting of human stem cell derived tissue into adult mice is another exciting new development [23, 24] but the influence of the murine host on the human transplant needs to be addressed further.

To study α-synuclein (αS) inclusion formation, we induced seeding in long-term murine hippocampal slice cultures obtained from postnatal mouse brain, which preserves the complex cellular brain microenvironment. We found inclusions in both neurons and microglia with spreading of the lesions along neural pathways. Both induction and propagation could be blocked by a human αS seed-recognizing antibody. To foster translation, progression of synucleinopathy and associated neurodegeneration in these cultures was monitored by assessing neurofilament light chain (NfL) protein levels in the culture medium, a biomarker used in preclinical animal studies as well as in clinical settings. We then applied the initial findings from the murine cultures to resection-derived adult human brain tissue cultures and succeeded in inducing αS inclusions in a true adult human brain environment.

## Methods

### Mice

Wildtype C57BL/6 J, heterozygous Thy1-h[A53T]αS transgenic (tg) [25], and Snca^−/−^ (C57BL/6-Snca^*tm1Rosl*^) [26] pups were used for preparation of HSCs. Thy1-h[A53T]αS tg mice overexpress human mutant (A53T) αS under control of a Thy1-promoter element. Experimental procedures were carried out in accordance with the veterinary office regulations of Baden-Wuerttemberg (Germany) and were approved by the local Animal Care and Use Committees.

### Preparation of mouse hippocampal slice cultures (HSCs)

HSCs were prepared from pups at postnatal day 4–6 (P4–6) according to previously published protocols [27, 28]. After decapitation, brains of the pups were aseptically removed, the hippocampi were dissected and cut perpendicular to the longitudinal axis into 350 μm sections with a tissue chopper. Carefully selected intact hippocampal sections were transferred into petri dishes containing an ice-cold buffer solution (minimum essential medium (MEM) supplemented with 2 mM GlutaMAX™ at pH 7.3). Three sections were placed onto a humidified porous polyethylene (PTFE) membrane insert (Merck Millipore, PICM0RG50) and into a 6-well plate with 1.2 ml culture medium (20% heat-inactivated horse serum in 1x MEM complemented with GlutaMax™ (1 mM), ascorbic acid (0.00125%), insulin (1 μg/ml), CaCl_2_ (1 mM), MgSO_4_ (2 mM) and D-glucose (13 mM) adjusted to pH 7.3) per well. HSCs were kept at 37 °C in humidified CO_2_-enriched atmosphere. The culture medium was completely changed three times per week.

### Preparation and treatment of human slice cultures

 Approval (#338/2014BO2) of the ethics committee of the University of Tuebingen as well as written informed consent was obtained from all patients and allowed spare tissue from resective brain surgery to be used for human slice cultures. Human neocortical slice cultures were prepared according to a previously published protocol [29]. Access tissue from temporal lobe was obtained from patients who had undergone resective brain surgery (patient 1: 61 years old, female, epilepsy due to ganglioglioma; patient 2: 49 years old, male, epilepsy due to cortical tubers; patient 3: 22 years old, male, epilepsy due to hippocampus sclerosis). Tissue was transported from the surgery room to the laboratory in oxygenated (95% O_2_ and 5% CO_2_) ice-cold artificial cerebrospinal fluid (aCSF; 110 mM C_5_H_14_ClNO, 26 mM NaHCO_3_, 10 mM D-glucose, 11.6 mM Na-ascorbate, 7 mM MgCl_2_, 3.1 mM Na-pyruvate, 2.5 mM KCl, 1.25 mM NaH_2_PO_4_, and 0.5 CaCl_2_). The tissue was cut into 250 μm slices perpendicular to the cortical surface using a vibratome. Afterwards, the slices were kept in aCSF equilibrated with carbogen for 30 min at room temperature (RT) before they were transferred onto culture membrane inserts (Merck Millipore, PICM0RG50) in a 6-well plate for cultivation. For the first hour following the slicing procedure, the slices were cultured in 1.5 ml NSC media (48% DMEM/F-12 (Life Technologies), 48% Neurobasal (Life Technologies), 1x N-2 (Capricorn Scientific), 1x B-27 (Capricorn Scientific), 1x Glutamax (Life Technologies), 1x NEAA (Life Technologies) + 20 mM HEPES. From then on, human slice cultures were grown in human cerebrospinal fluid (hCSF) obtained from normal pressure hydrocephalus patients via lumbar puncture [29]. ﻿ Approval of the ethics committee of the University of Tuebingen as well as written informed consent from all patients was obtained (#338/2016A). The hCSF was collected, pooled and centrifuged at 2,000 x g at 4 °C for 10 min. The supernatant was kept at − 80 °C and thawed at RT before changing the medium. Cultures were kept in 1.5 ml hCSF at 37 °C in humidified CO_2_-enriched atmosphere. The hCSF was completely changed three times per week. For αS overexpression, AAV1/2-CMV/CBA-human-A53T-α-synuclein-WPRE-BGH-polyA virus (AAV-hA53T-αS) (Vigene Biosciences, titer of 5 × 10^12^) was injected evenly spaced, once per 5 mm^2^ of the slice, using a picospritzer (PDES-O2DX/NPI electronics, Tamm, Germany).

### Brain-derived αS seeds and αS pre-formed fibrils

For brain-derived seeds, brain homogenates were prepared as previously described [13]. In short, fresh frozen pooled brainstems from spontaneously ill male and female Thy1-h[A53T]αS tg mice (7–8 months old, *n* = 3 brainstems per homogenate) and corresponding non-tg controls (20 months old, *n* = 2) were used. Homogenisation at 10% (w/v) was done in sterile PBS (Pre-cellys, 4 × 10 s at 5500 rpm), followed by vortexing and centrifugation at 3,000 x g for 5 min at 4 °C. The supernatants were collected, aliquoted and stored at −80 °C until use.

Expression in *E. coli*, purification and quality control of human recombinant monomeric wt αS was done as previously described [30]. For fibril formation, soluble wt αS was incubated in Tris-HCl buffer (50 mM Tris-HCl, pH 7.5, 150 mM KCl) at 37 °C under continuous shaking for 5 days and formation of fibrils was assessed with Thioflavin T. The fibrils were quality checked by transmission electron microscopy after negative staining before and after fragmentation as reported previously [30, 31]. In addition, the fibrils limited proteolytic pattern was also assessed as previously reported [30, 31]. Fragmentation was achieved by sonication using a sonotrode (sonication for 20 min, 0.5 s pulses; Sonicator UIS250V, equipped with VialTweeter, Hielscher Ultrasound Technology, Germany). The fibrils were imaged using a Jeol 1400 transmission electron microscope following their adsorption onto carbon-coated 200 mesh grids and negative staining with 1% uranyl acetate. The images were recorded using a Gatan Orius CCD camera (Gatan Inc., Pleasanton, CA, USA) (Supplementary Fig. 1). The average size of the fibrils after fragmentation (47 ± 5 nm) was derived from length distribution measurements and their average molecular weight (16,200 kDa) was derived from analytical ultracentrifugation sedimentation velocity measurements. The fibrils (350 μM) were aliquoted (6 μl per tube), flash frozen in liquid nitrogen and stored at − 80 °C. Fibrils were labeled with NHS-ester ATTO-550 as previously described [32].

### Seeding of the cultures

Murine HSCs were kept for 10 days in vitro (DIV-10) without any experimental treatment. At DIV-10, 1 μl of αS pff (350 μM or dilutions thereof) or brainstem homogenate of Thy1-h[A53T]αS tg or wt mice was pipetted on top of each culture. Human slice cultures were kept until DIV-3 without any experimental treatment. At DIV-3, 1 μl of αS pff (35 μM) was pipetted on top of the culture.

Local injection of αS pff into HSCs was performed into cornu ammonis 3 (CA3) at DIV-10. CA3 was identified as the region next to dentate gyrus (DG), which can be observed by light microscopy (dense cell layer resembling a horseshoe). Microinjection pipette (Science Products GmbH, GB150TF-10) was pulled using a micropipette puller (Sutter instruments, Model P-97; settings: 1 cycle, heat = 520, pull = 50, velocity = 50, time = 250). To immediately visualise the injection, 0.3 μl FastGreen dye (Carl Roth) was added to a 6 μl aliquot of ATTO-550-labelled or unlabelled αS pff (350 μM), upon loading the pipette. The loaded pipette was then inserted into the holder of a picospritzer (PDES-O2DX/ NPI electronics, Tamm, Germany), and the very end of the tip was carefully broken under visual guidance with sterile forceps, until a pressure pulse of 10 ms was able to release a small droplet of αS pff from the tip. Post-injection microscopic examination of the broken tip revealed an opening size of 25–40 μm. HSCs in their 6-well-plates were placed under the light microscope, and the pipette was carefully inserted into CA3. With a pressure pulse of 10 to 20 ms (10 ms for larger tip openings, 20 ms for smaller tip openings), a small volume of αS pff was injected. After having injected all slices, (approximately 1 h later) the medium was changed.

### Antibody treatment

The antibody HLu-3 is a human IgG1-recognizing αS. The epitope of the antibody was determined to be amino acid 113–115 of human αS, using arrays of overlapping linear peptides at Pepscan (Pepscan Zuidersluisweg 28,243 RC Lelystad, The Netherlands). The affinity to human, mouse and cynomolgus αS monomers is determined to be 31 nM by surface plasmon resonance (BIAcore® 3000). HLu-3 has approximately a 200-fold avidity shift in the binding to fibrillated forms of human αS (determined by competition ELISA). HLu-3 has no cross-reactivity to beta- & gamma-synuclein. Reference item used in the study was a negative isotype-matched control, anti-HIV-1 gp120 human IgG1 antibody (b12).

HLu-3 or control antibody were either mixed with human αS pff (1:1 in PBS to obtain a final concentration of 35 μM) and drop seeded on top of each HSC at DIV-10, or added to the medium. For the latter, antibodies were added to pre-warmed culture medium upon medium change (final concentration of 350 nM). Depending on experimental scheme, antibody-supplemented medium was used 7 days prior to the application of αS pff seeds (-7 days post-injection, dpi) or 1 h after αS pff seeds (0 dpi). Thereafter, the cultures were treated with antibody-supplemented medium with every medium change.

### Histological analysis of cultures

Cultures were fixed with 4% paraformaldehyde (PFA) in PBS at pH 7.4 for 2 h (HSCs) or for 24 h (human slice cultures). After fixation, HSCs were rinsed 3 times with 0.1 M PBS for 10 min and stored in PBS at 4 °C for up to 1 month until sectioning. Human slice cultures were rinsed 3 times with PBS supplemented with 0.2% TritonX-100 (PBST) and incubated with PBST overnight for permeabilization. The membrane carrying the fixed HSCs was cut out and mounted onto a planar agar block. With a vibratome (Leica VT 1000S Vibratome, Leica Bio-systems), the cultures were sliced into 50 μm sections. Typically, 5–6 intact sections per HSC were obtained and collected in PBS to be stained within 1 week. Human slice cultures were stained unprocessed.

Antigen retrieval was performed by heating the sections in 10 mM citrate buffer (1.8 mM citric acid, 8.2 mM trisodium citrate, pH 6.0) at 90 °C for 35 min (HSCs) or overnight at 4 °C and subsequently 30 s at 90 °C (human slice cultures). Sections were blocked with 5% NGS (HSCs, 2 h) or 1% NGS (human slice cultures, overnight at 4 °C) and 0.3% PBST. For detection of αS phosphorylated at Ser-129, a rabbit monoclonal pS129 antibody (Abcam, EP1536Y, Cat# ab51253, 1:1,000) was used. For microglia detection rabbit monoclonal iba1 (Wako Chemicals GmbH, Cat# 019–19,741, 1:250) and for neuronal staining and structure mouse monoclonal NeuN antibody (Millipore GmbH, Cat# MAB377, 1:500) and chicken anti-MAP2 (Abcam, Cat# ab5392, 1:500) were used. Following Alexa-fluorophore-conjugated secondary antibodies were applied in a concentration of 1:250: goat-anti-rabbit Alexa-568 (Thermo Fisher, Cat# A11011); goat-anti-mouse Alexa-488 (Thermo Fisher, Cat# A11001); goat-anti-mouse Alexa-568 (Invitrogen, Cat# A11004); goat-anti-rabbit Alexa-633 (Thermo Fisher, Cat# A21070); goat-anti-chicken Alexa-488 (Invitrogen, Cat# A21467). DAPI counterstaining was performed at a concentration of 1:500.

For staining with amyloid binding dyes pentamer formyl thiophene acetic acid (pFTAA) [33, 34] and thioflavin S (ThioS), sections were incubated for 1 h with either freshly prepared pFTAA (1.5 mM in de-ionized water, used at 1:500 in PBS) or ThioS (Sigma-Aldrich, Cat# T1892; 1% w/v ThioS in milliQ H_2_O). ThioS-stained sections were washed 2 x in 70% EtOH and for 10 min. Slices were transferred on glass slides and coverslipped with Dako Fluorescence mounting medium (Biozol Diagnostika, Cat# S3023).

Sections were analyzed using an Axioplan2 imaging microscope (Zeiss, Jena, Germany) and digitised with an AxioCam HRm black and white camera (Zeiss) using AxioVision 4.8 software (Zeiss). With a Plan Neofluar 10x/0.50 objective lens (Zeiss), 16-bit RGB mosaics of the whole culture were obtained with a resolution of 170 pixels / μm. High resolution images were acquired using a Zeiss LSM 510 META (Axiovert 200 M) confocal microscope with an oil immersion 40 × /1.3 or 63 × /1.4 Plan Apochromat objective and LSM software 4.2 (Carl Zeiss). Sequential excitation of fluorophores was performed using lasers with the wavelength 405 nm (DAPI), 488 nm (Alexa-488 coupled secondary antibodies, ThioS, pFTAA), 543 nm (Alexa-568 coupled secondary antibodies, pFTAA), and 633 nm (Alexa-633 coupled antibodies).

### Quantification of immunohistochemical stainings

For quantification of pS129- and ThioS-positive inclusions in HSCs, whole culture mosaic images were acquired on an Axioplan2 imaging microscope as described above. Cultures with sectioning artefacts or cultures that were injected in the wrong site (e.g. into CA1 instead of CA3) were excluded from the analysis with FIJI ImageJ (version 2.1.0/1.53c). Images were blinded, colour channels were split, background was subtracted (rolling ball radius 50 pixels), and the intensity threshold was manually adjusted. To exclude unspecific staining of ThioS, the particle size of signal in the green channel was limited to 20–200 μm^2^. On each mosaic, the percentage of pS129- and ThioS-positive signal over the whole culture was calculated.

To quantify percentage of pS129 and ThioS-signal in the injection site and other hippocampal subregions, images were blinded, regions of interest (ROIs) for CA3, CA2, CA1, DG and subiculum were selected based on the brain map of the Allan Mouse Brain Atlas (2004) (P56, coronal) and stored in FIJI before channels were split. Background was subtracted and intensity threshold for pS129 or ThioS was adjusted for the whole culture. For each ROI, the percentage of pS129- and ThioS- positive area over the respective ROI area was calculated.

### Heatmaps

To demonstrate spreading through hippocampal subregions, heatmaps were prepared by illustrating the mean percentage of pS129- or ThioS area obtained in the ROI analysis (see “Quantification of immunohistochemical stainings”) by a colour code. Using Microsoft Excel (v.16), mean values were assigned to a colour based on a 3-colour-scale from white (#FFFFFF) via yellow (#FDBF2D) to red (#BE0712). The colours were identified using Adobe Photoshop CS5 (v.12) and applied to the respective area of a hippocampal map in Adobe Illustrator CS5 (v.15).

### Biochemical analysis of cultures

Slice culture homogenates were prepared from treated or untreated cultures. Cultures were removed from the membrane, pooled (*n* = 16) and immediately frozen on dry ice and stored at − 80 °C until use. Frozen slice cultures were homogenised with a syringe in 160 μl sterile PBS, aliquoted and stored at − 80 °C until further use. For immunoassays (Western blotting) homogenates in PBS were shifted to high salt (HS) buffer (50 mM Tris-HCl pH 7.5, 750 mM NaCl, 5 mM EDTA, 1% phosphatase and protease inhibitor cocktails). 100 μL of homogenate were incubated on ice in *N*-lauroylsarcosyl (Sigma, Cat# 61747, Saint-Quentin-Fallavier, France) at a final concentration of 10%, and were left on ice for 15 min before they were loaded on a 10% sucrose cushion and ultracentrifuged at 186,000 x g for 1 h 10 min at 4 °C. The supernatant was collected, and the pellet was resuspended in sample buffer, and sample buffer was also added to the supernatant. Proteins were separated in a 4–12% SDS NuPage Gel and electroblotted onto Amersham nitrocellulose membranes (VWR International Merck Eurolab, Cat# 10600001). Membranes were incubated with 0.4% PFA for 30 min, and then saturated with 5% dry milk in 0.1% PBS-Tween20 (0.1%). Monoclonal rabbit antibody against pS129 (AbCam, Cat# ab51253) at 1:1,000 dilution to detect phosphorylated αS species, monoclonal mouse antibody against αS (BD Transduction Laboratories, Cat# 610786) at 1:1,000 dilution to detect total αS, and rabbit β-actin antibody (AbCam, Cat# ab8227) as a loading control were used. Membranes were then incubated for 1 h with anti-rabbit antibody or anti-mouse antibody at 1:20,000 for 1 h at RT. Samples were visualized with chemiluminescence using SuperSignal West Dura Extended or Pico (both Thermo Scientific).

### Immunoassay for total αS measurements in brain homogenate

For αS measurements, brain homogenates were extracted as follows: aliquots were thawed on ice, mixed 1:3.2 with cold formic acid (FA) (minimum 96% purity; Sigma, St. Louis, MO, USA), sonicated for 35 s at 4 °C, and spun at 25,000 g at 4 C for 1 h. The supernatant was equilibrated (1:20) in neutralization buffer (1 M Tris base, 0.5 M Na_2_HPO_4_, 0.05% NaN_3_).

Concentrations of human αS in brain homogenates were determined with an electrochemiluminescence-linked immunoassay using the MSD Human α-Synuclein Kit (Meso Scale Discovery, Gaithersburg, MD, USA), or by Single Molecule Array (Simoa) technology using the Simoa™ Human Alpha-Synuclein Discovery Kit (Quanterix, Billerica, MA, USA) according to manufacturer’s instructions. FA-soluble brain homogenates were diluted up to 1:10,000 in Diluent 35 (Meso Scale Discovery) or 1:100 in Alpha-Synuclein Sample Diluent (Simoa) before the measurement, and analyzed in duplicates on a Mesoscale Sector Imager 6000 or a Simoa HD-1 Analyzer. MSD DISCOVERY WORKBENCH software 3.0 or Simoa Software Version 1.5 for HD-1 Analyzer was used for data analysis. Internal reference samples were used as controls on every plate.

### Immunoassay for aggregated αS measurements in brain homogenate and pre-formed fibrils

αS aggregates were measured using a HTRF-FRET assay developed by Cisbio (#6FASYPEG, Cisbio). αS pff and tg brain homogenate were serially diluted and a HTRF-FRET signal measured on a PHERAstar (BMG LABTECH) using 337 nm laser excitation, simultaneous dual emission 665 nm / 620 nm and HTRF technology. Data is reported as 665 nm / 620 nm x 10,000. αS pff needed to be diluted > 50,000 fold to be on the proper side of the hook effect of the assay, whereas the tg brain homogenate did not show hook effect issues at any dilutions. For αS pff, a dilution of 204,800-fold resulted in a signal of approximately 20,000, whereas the tg brain homogenate only was diluted 25-fold to reach a similar aggregation level. The difference in dilutions (e.g. 204,800-fold vs 25-fold) to reach a similar aggregation level was used to estimate the relative αS aggregation level per volume of sample. Results over several dilutions were combined to reach the final result of 8,000 x more aggregates in αS pff relative to tg brain homogenate per volume.

### NfL immunoassay

﻿Culture medium was collected, aliquoted, and kept at −80 °C until use. NfL concentrations were determined by Single Molecule Array (Simoa) technology using the highly sensitive Simoa™ NF-Light Advantage Kit (Quanterix, Billerica, MA, USA) according to manufacturer’s instructions [35]. Medium samples were pre-diluted 1:10 or 1:50 in NF-Light sample diluent and measured in duplicates on a Simoa HD-1 Analyzer (Quanterix). Internal reference samples were used as controls on every plate. Detection limit of NfL was 0.038 pg/ml.

### Culture thickness

While sectioning the freshly fixed cultures with a vibratome (see above), the amount of 50 μm sections was assessed. Although the first and the last section sometimes were not exactly 50 μm, the amount of sections × 50 μm equals roughly the culture thickness at the time of fixation.

### Intracerebral injection of αS tg mice

Male and female 3–4-month-old Thy1-h[A53T]αS tg mice were anesthetized using a mixture of ketamine (100 mg/kg body weight) and xylazine (10 mg/kg body weight) in saline. 2.5 μl of brain homogenate (see above) was then infused bilaterally into the dorsal hippocampus (AP − 2.5 mm, L ± 2.0 mm, DV − 1.8 mm) by stereotactic injection. Injection speed was 1.25 μl/min, and the needle was slowly removed after being kept in place for an additional 2 min. The surgical area was cleaned with sterile saline and the incision was sutured. Mice were kept under infrared light for warmth and monitored until recovery from anaesthesia.

After incubation periods of up to 30 days, mice were perfused for 5 min with ice-cold PBS. Brains were removed and immersion-fixed in 4% PFA in 0.1 M PB at pH 7.4 for 48 h, and then placed in 30% sucrose in PBS for 48 h. Brains were frozen in 2-methylbutane, cooled with dry ice, and then serially cut into 25 μm sagittal sections using a freezing-sliding microtome. The sections were collected in cryoprotectant (35% ethylene glycol, 25% glycerol in PBS) and stored at − 20 °C until use. Immunohistochemical staining of αS inclusions was done as described for HSCs (see above).

### Statistical analysis

All statistics were performed using PRISM software (GraphPad v.9). Data were tested for normality using the Shapiro-Wilk test. If the groups passed the normality test and only two groups were compared, an unpaired two-tailed t-test was applied. If the two groups did not pass the normality test, the non-parametric Mann-Whitney test was used for comparison. To estimate the influence of two different variables, a two-way-ANOVA was performed. If ANOVA revealed significant effects, *post hoc* Bonferroni’s multiple comparisons test was used. The mean and standard error of the mean (SEM) are reported for each experimental group.

## Results

### Induction of αS inclusions in murine hippocampal slice cultures

**Fig. 1:**
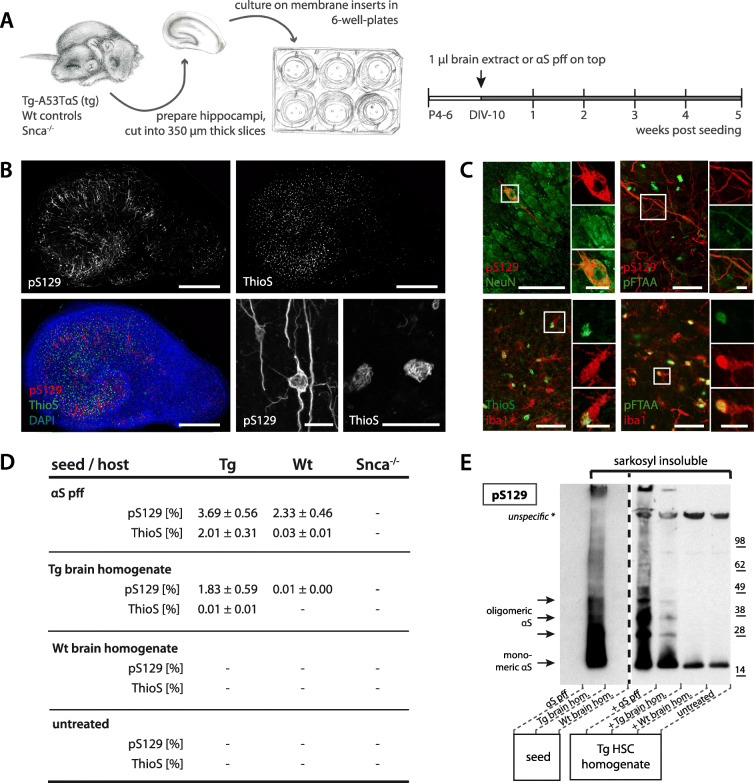
Induction of αS inclusions in mouse hippocampal slice cultures. **(A)** Treatment scheme for hippocampal slice cultures (HSCs). Hippocampi of postnatal day 4–6 (P4–6) of Tg-Thy1-h[A53T]αS (Tg), wildtype (Wt) or *Snca*^−/−^ mice were sectioned into 350 μm thick slices and plated onto semi-permeable culture membranes. After 10 days in vitro (DIV-10), 1 μl of either Wt or Tg brain homogenate, or αS preformed fibrils (αS pff) at 35 μM were added on top of each culture. All three cultures within one well received the same treatment. Five weeks later, the cultures were collected for analysis. **(B)** Shown is a Tg culture treated with αS pff. Anti-pS129 immunofluorescence revealed a myriad of inclusions in both somas and processes of cells with a neuronal appearance. In contrast, ThioS staining revealed mainly small wool-like inclusions. Shown is also the merged triple staining with DAPI. Scale bars represent 500 μm (overview), and 20 μm (closeup). **(C)** Immunofluorescence double staining for pS129 (red) and NeuN or pFTAA (green) as well as for iba1 (red) and ThioS or pFTAA (green). Note that pS129-positive inclusions are predominantly present in neurons (NeuN), while ThioS-positive aggregates appeared mainly to be associated with microglia (iba1) consistent with previous work [36]. pFTAA stained both pS129-positive neuritic inclusions and microglial inclusions. Scale bars represent 50 μm, and 10 μm for inserts. **(D)** Quantification of pS129- and ThioS-positive inclusions (in percentage of culture area) in dependence of the seeding agent (αS pff, Tg brain homogenate) and host (Tg, Wt, Snca^−/−^) 5 weeks after seeding. Mean ± SEM is shown; *n* = 5–6 HSCs per group; − = no inclusions. **(E)** Immunoblot using an antibody against pS129 of sarkosyl-insoluble material of seeds and HSC homogenates. Monomeric αS phosphorylated at S129 was present as a band at around 15 kDa in brain extract as well as tg HSCs seeded with αS pff or Tg brain homogenate, but not αS pff. Multimeric species were also present as higher molecular bands; *n* = 16 pooled HSCs per group

Murine hippocampal slice cultures (HSCs) were prepared from postnatal days 4–6 Thy1-h[A53T]αS tg or wt mice (tg HSCs vs wt HSCs) and grown in culture for 10 days to stabilize. αS inclusions were induced by one-time application of αS seed-rich aged Thy1-h[A53T]αS transgenic (tg) mouse brain homogenate or alternatively by synthetic αS preformed fibrils (pff) (Fig. 1A). Five weeks later, all cultures had developed abundant perikaryal and neuritic αS lesions stained with an antibody specific for phosphorylation at serine 129 (pS129) (Fig. 1B and C), a surrogate marker of neuronal αS assembly [37]. The neuronal αS inclusions stained also positive for the amyloid-binding dye pFTAA, but much less so for Thioflavin S (ThioS). However, there were numerous ThioS-positive inclusions that were largely localized to microglia (Fig. 1C), consistent with recent observations in αS tg mice [36]. The microglial inclusions were always in vicinity of the neuronal inclusions. They appeared wool-like and were localized mainly close to the nucleus with an overall appearance different from the neuronal inclusions (Fig. 1B and C). For both neuronal and microglial lesions, the induction was faster and more abundant in tg HSCs compared to wt HSCs (Fig. 1D). Untreated control cultures, cultures treated with wt brain homogenate, and Snca^−/−^ cultures did not develop any αS inclusions (Fig. 1D; Supplementary Fig. 2). Analysis of the sarkosyl-insoluble HSCs fractions of both αS pff and tg brain homogenate-treated cultures confirmed the presence of aggregated αS very similar to that in the brain homogenates (Fig. 1E).

### Induction of αS inclusions is dependent on seed concentration and incubation time

**Fig. 2:**
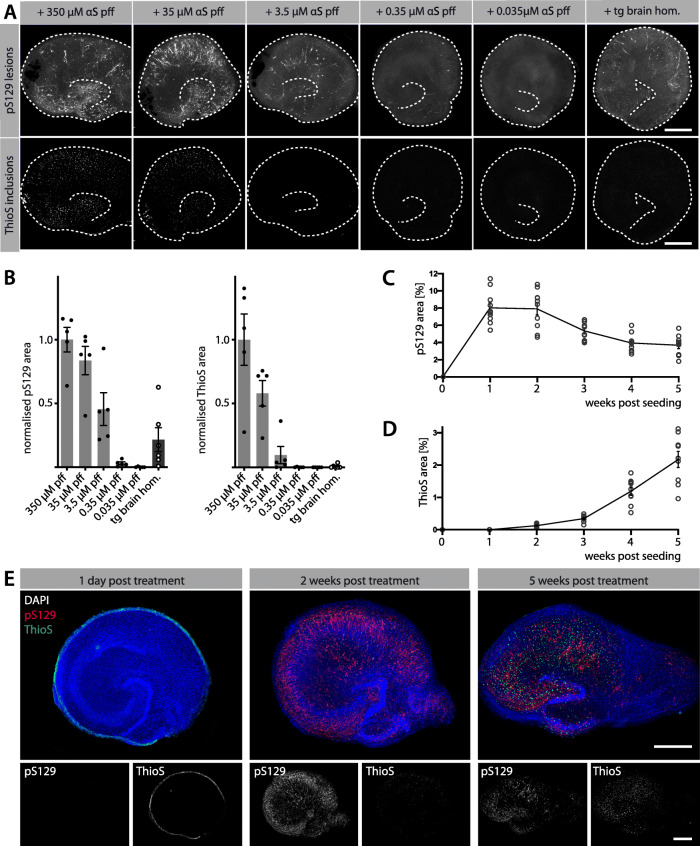
Induction of αS inclusions is seed-, concentration- and time-dependent. See Fig. 1A for treatment scheme. **(A)** Immunofluorescence staining for pS129 and ThioS in tg HSCs at 5 weeks post-seeding. The concentration of αS pff (350 μM with subsequent 10- to 10,000-fold dilutions) determines the abundance of pS129-positive αS inclusions (largely neuronal) and ThioS-positive inclusions (largely microglial) in tg HSCs. Brain homogenate shows approximately the seeding activity of 1:100 αS pff. However, the seed concentration in the brain homogenate is likely much lower compared to the one of αS pff (see Results). Scale bars = 50 μm. **(B)** Normalised quantification of pS129-positive and ThioS-positive inclusions in tg HSCs treated with different concentrations of αS pff (light grey bars) or with tg brain homogenate (dark grey bar). Mean ± SEM is shown; *n* = 5–6 HSCs per group. **(C, D)** Quantification of percentage of pS129-positive (C) and ThioS-positive (D) αS inclusions over time using 35 μM αS pff. Mean ± SEM are shown; *n* = 9 HSCs per group and timepoint. **(E)** Distribution of pS129- and ThioS-positive aggregates in tg HSCs treated analysed at 1 day, 2 weeks or 5 weeks post-treatment. After 1 day, ThioS-positive αS pff (green) still surrounded the culture with no induction of αS lesions. At 2 weeks, pS129-positive neuronal inclusions (red) were abundant, but appeared to decline at 5 weeks post-seeding. In contrast, ThioS-positive microglia inclusions increased from 2 to 5 weeks post-seeding. Scale bars represent 500 μm

Titration experiments revealed increased neuronal and microglial inclusions with increasing concentration of the seeds (Fig. 2A, B). Tg brain homogenate appeared approximately 100 x less potent (Fig. 2B) but also contained 1,000 x fewer monomeric αS equivalents (typically 5 μg/ml; measured by immunoassays after formic acid treatment) compared to undiluted αS pff (350 μM or 5 mg/ml). This observation suggests that tg brain-derived αS seeds are more seeding potent than their synthetic counterparts, which is also in line with direct measurements of αS aggregates (Supplementary Fig. 3).

Neuronal αS lesions appeared in tg HSCs after 1 week and decreased slightly after 2 weeks (Fig. 2C). Interestingly, microglial inclusion appearance was delayed and occurred first after 2 weeks and increased thereafter (Fig. 2D, E). To investigate the temporal appearance of αS lesions in vivo*,* tg brain extract seeding in adult mice of the same genotype (Thy1-h[A53T]αS tg mice) was performed. Here, we observed an increasing quantity of lesions which are not reaching to plateau within 30 days attributed to the fewer seeds in tg brain extract (Supplementary Fig. 4).

### αS lesion-related neurodegeneration can be monitored by NfL release in culture medium

**Fig. 3:**
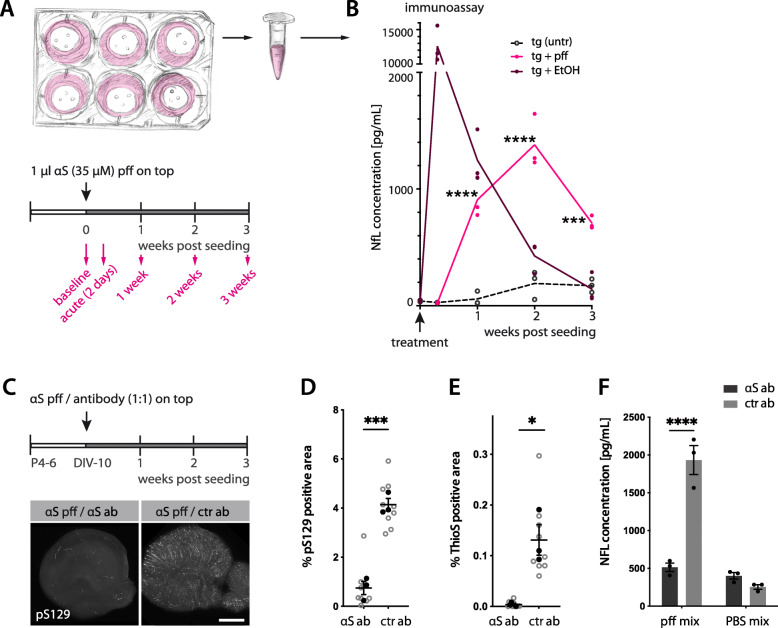
Increased Neurofilament light chain (NfL) in the culture medium in response to the induction of αS lesions. **(A)** Schematic of a well-plate containing HSCs and medium collection scheme. Each well contains 3 cultures of the same treatment. The entire culture medium (1.2 ml) of each well was collected (and immediately replaced with new one) before seeding (baseline), 2 days (acute), and 1, 2, and 3 weeks after seeding. **(B)** Longitudinal measurement of NfL in culture medium of tg HSCs measured by immunoassay. Cultures were treated with 35 μM αS pff (pink), or left untreated (black, dotted line). As a control, cultures were also treated with EtOH (dark red). While EtOH treatment had an acute toxic effect, αS pff treated cultures released NfL gradually into the medium with an apparent peak at 2 weeks post-seeding. Mean ± SEM is shown; *n* = 3 wells (each containing 3 cultures) per group; two-way-ANOVA with repeated measurements (treatment F(1, 4) = 79.86, *p* = 0.0009; time F(3, 12) = 78.57, *p* < 0.0001; interaction F(3, 12) = 51.69) with Bonferroni’s corrections for multiple comparisons between treatment groups and within time points (***p < 0.0001). The experiment was repeated with a new batch of αS pff and resulted in somewhat higher NfL levels (171.0 ± 136.8, 2453.7 ± 169.7, 3800.0 ± 431.2 and 2713.7 ± 575.7 pg/mL for 2d, 1 wk., 2 wk., 3 wk., respectively) in comparison to the untreated (43.7 ± 6.6, 248.0 ± 89.5, 234.3 ± 16.6 and 158.3 ± 24.3 pg/mL for 2d, 1 wk., 2 wk., 3 wk., respectively; *n =* 3 wells (each containing 3 cultures) per group. **(C-F)** αS seed inactivation blocks NfL increase in culture medium. 35 μM αS pff were mixed 1:1 with 35 μM αS- or ctr-antibody before applying onto each tg HSC. After 3 weeks, αS pff / αS antibody-treated HSCs showed very little pS129-positive inclusions compared to αS pff / ctr antibody treated HSCs (C). Scale bar = 500 μm. Quantification of pS129-positive inclusions (D, unpaired two-tailed t-test, t(4) = 9.22, ***p = 0.0008) and ThioS-positive inclusions (E, unpaired two-tailed t-test, t(4) = 4.19, *p = 0.0138). Mean ± SEM is shown with *n =* 3 wells (each containing 3 cultures and the mean of the three cultures (black dots) was taken as one data point; grey dots are values for each culture). NfL measurements in the culture medium of αS pff / antibody mix and corresponding PBS-treated cultures (i.e. with no induction of αS lesions) is shown at 2 weeks post-treatment time points (F). Mean ± SEM; *n =* 3 wells, two-way ANOVA for treatment (pff vs PBS) F(1, 8) = 75.64, *p <* 0.0001; antibody F(1, 8) = 38.16, *p* = 0.003; interaction F(1, 8) = 57.850, *p <* 0.0001). Bonferroni’s correction for multiple comparisons revealed ****p = 0.0001 for αS vs ctr antibody

A sensitive measure for neurodegeneration is the thinning of the cultures. A clear degenerative phenotype was observed in tg HSCs with the highest concentration of αS pff treatment (350 μM) (Supplementary Fig. 5). To assess the extent of subtler neurodegeneration, the release of neurofilament light (NfL) into the culture medium was measured. Increased levels of NfL have previously been found in CSF and blood of Thy1-h[A53T]αS tg mice and has been linked to the induction and spreading of αS lesions [38]. Indeed, an increase in NfL upon induction of αS pathology was also found in tg HSCs treated with 35 μM αS pff (Fig. 3A, B).

A link between the induced αS pathology and the increase in NfL release was demonstrated by blocking αS lesion formation. Three weeks -after mixing and treating cultures with human anti-αS antibody and αS pff, analysis revealed greatly reduced neuronal and microglial inclusions (Fig. 3C - E) and a concomitant attenuation of the NfL concentration in the culture medium (Fig. 3F).

### Seed-induced synucleinopathy propagates to interconnected regions

**Fig. 4:**
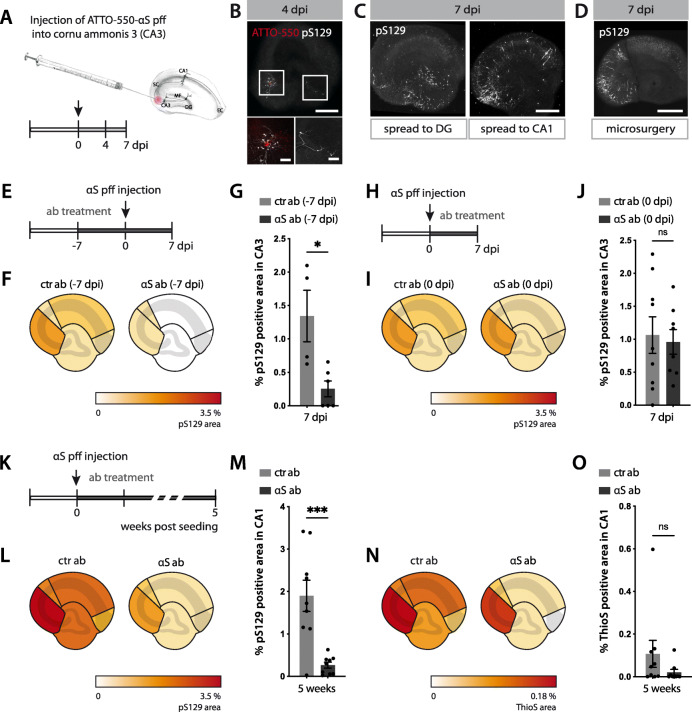
Spreading of αS lesions in mouse hippocampal slice cultures. **(A)** Schematic illustration of the local injection paradigm of ATTO-550-labelled αS pff (from the 35 μM stock solution) into hippocampal CA3 region. **(B)** ATTO-550-αS pff (red) and the first neuritic pS129-positive inclusions (white) at the injection site (CA3) and in the dentate gyrus (DG) at 4 dpi. Note that αS pff seeds are not phosphorylated upon uptake [32, 39] and therefore the pS129-positive signal can be attributed to aggregated endogenous αS. Scale bars = 500 μm and 100 μm (inserts). **(C)** Apart from the injection site (CA3) and DG, pS129-positive inclusions also appeared in CA1 at 7 dpi. Scale bars = 500 μm. **(D)** Disconnecting CA3 from both CA1 and DG via microsurgery before injecting pff inhibited the spreading of the pathology at 7 dpi. Scale bars = 500 μm. **(E-G)** Antibody-mediated blocking of αS lesion induction. Schematic illustration of experimental setup (E). Seven days prior to αS pff injection into CA3, anti-αS or ctr antibodies were added to the medium (reaching a final concentration of 350 nM) and antibody treatment was continued until 7 dpi. Heatmap of pS129-positive area revealed largely reduced pS129-positive inclusions (F). Quantification of pS129-positive inclusions in CA3 (G). Mean ± SEM; *n* = 6 and 4 HSCs/group for the αS and ctr antibody, respectively (2 HSCs from ctr group were excluded due to accidental injection into CA1 region); unpaired two-tailed t-test (t(8) = 3.211, *p* = 0.0124, *p < 0.05). **(H-J)** Delayed antibody-treatment fails to block αS lesion induction. Schematic illustration of experimental setup (H). Antibodies were added to the culture medium 1 h after αS pff injection into CA3 and antibody treatment was continued until 7 dpi. Heatmap revealed that such delayed antibody treatment did not prevent pS129-positive inclusions in injection region (I). Quantification of pS129-positive inclusions in CA3. Mean ± SEM; *n =* 9 HSCs/group; unpaired two-tailed t-test (t(14) = 0.7102, *p* = 0.4892). **(K-O)** Antibody-mediated blocking of the spreading of the αS lesion. Schematic illustration of experimental setup (K). Antibodies were applied 1 h after αS pff injection into CA3 and antibodies were continuously added until 5 weeks post-seeding. Heatmap revealed that spreading of pS129-positive inclusions to other subregions has been largely reduced (L) and the same was true for ThioS-positive inclusions (that start to develop 2–3 weeks after the neuronal inclusions, see Fig. 2) (N). Quantification of pS129-positive inclusions (M) and ThioS-positive inclusions (O) in CA1. Mean ± SEM; *n =* 9 HSCs/group; for pS129 (unpaired two-tailed t-test (t(16) = 4.362, ***p = 0.0005); for ThioS**,** two-tailed Mann-Whitney test (U = 25, *p* = 0.1782)

To establish HSCs as a model system to study the propagation of αS aggregates, ATTO-550-labelled αS pff were injected into the CA3 region (Fig. 4A) in order to observe whether locally induced αS inclusions spread to the distant CA1 via Schaffer collaterals and/or retrogradely via mossy fibres to the dentate gyrus (DG). Examination revealed the formation of pS129-positive inclusions in neurites around the CA3 injection site already at day 4 with some early spreading to the DG (Fig. 4B). At 7 days post-injection, both DG and CA1 neurons exhibited somatic αS inclusions with neuritic inclusions along the Schaeffer collaterals and mossy fibres (Fig. 4C), suggesting both anterograde and retrograde spreading [40].

To rule out the possibility of diffusion of the applied seeds from the injection area in CA3 to DG and CA1, Schaeffer collaterals and mossy fibres were surgically disconnected. As a result, virtually no spreading across the incision site was apparent (Fig. 4D). Presuming that a simple mechanic cut would not impair diffusion of seeds, these results indicate that the observed formation of αS inclusions in distal regions spreading occurs via neural connection.

### Spreading of αS inclusions can be blocked by anti-αS antibody

Our initial observation that the αS antibody blocks seeding once mixed with αS pff (see Fig. 3) urged us to study whether the antibody was also able to block seeding and propagation of αS lesions when present in the culture medium (Fig. 4E - O). When the medium was continuously supplemented with αS antibody starting 7 days before αS pff injections, there was a robust blocking of αS lesions 1 week after seed application (Fig. 4E - G). However, when αS antibody was added to the medium 1 h after injection of αS pff, no reduction of αS lesions were observed at day 7 (Fig. 4H – J).

We then assessed the ability of the αS antibody to prevent the spreading of pathology from the CA3 injection site throughout the culture. αS pff seeds were injected into CA3 and, again, 1 h later, the culture medium was continuously supplemented with αS antibody (Fig. 4K). Five weeks later, there was a decrease of neuronal pathology (Fig. 4L, M) suggesting an inhibiting effect of the αS antibody on the spreading, particularly to CA1. A decrease in microglial inclusions was also apparent, but did not reach statistical significance (Fig. 4N, O).

### Induction of αS inclusions in adult human brain slice cultures

**Fig. 5:**
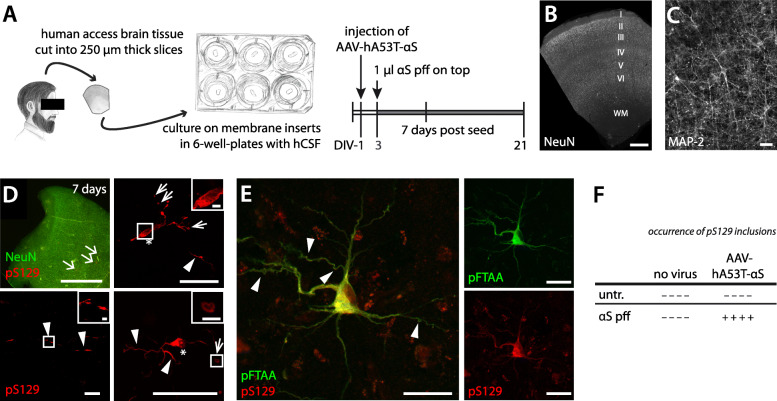
Induction of αS inclusion in adult human brain slice cultures. **(A)** Schematic of the preparation and treatment scheme of human neocortical slice cultures obtained from brain resection. Healthy access tissues of resected brain were used to prepare human slice cultures. Cortical tissue was sectioned into 250 μm slices perpendicular to the cortical surface. At day in vitro 1 (DIV-1), some cultures were injected with AAV-hA53T-αS to induce overexpression of αS. At DIV-3, 1 μl of αS pff (35 μM) was added on top of each culture. At DIV-10, the human slice cultures were fixed for histological analysis. Drawing shows fictional patient. **(B, C)** Immunofluorescence staining of neuronal nuclei (NeuN) show preserved neuronal layers I-VI at DIV-10, the time of fixation (WM = white matter) (B). Neuronal cell bodies with processes appear also intact (MAP-2-immunostaining; shown is DIV-12). Scale bars = 1 mm (B) and 50 μm (C). These observations are consistent with previous work demonstrating that resected human tissue can be cultured and maintain electrophysiological properties up to DIV-21 if the medium is replaced with human CSF [29]. **(D)** Immunofluorescence staining for pS129 (red) in a human slice culture at 7 days post-seeding. Arrows in top left panel point to sites where neurons developed pS129-positive inclusions. Neuronal nuclei were stained by NeuN (green). Top right, bottom left and bottom right panels show single neurons with pS129-positive inclusions (from 3 different cultures). Note the diffuse and large inclusion in the neuronal soma (asterisks) and the filamentous inclusions in the processes (arrowheads) with often large spheroid-like inclusions (narrow arrows). Scale bars represent 1 mm (top left), 50 μm (top right, and bottom row) and 5 μm (inserts). **(E)** Immunofluorescence staining for pS129 antibody (red) and the amyloid-binding dye pFTAA (green). Note that the red labelling in the background is lipofuscin autofluorescence. Scale bars represent 50 μm. **(F)** Table summarises the results from seeded αS aggregation in human cultures. The formation of pS129-positive inclusions is indicated by a (+) or (−) for each culture (*n* = 4 cultures/condition; cultures are derived from 3 patients with ages between 22 and 61 years old)

Having established the conditions needed to induce αS lesions in murine HSCs, we next addressed the possibility of adapting these findings to cultures derived from human adult brain tissue [29]. Such human cultures are derived from resected adult brain tissue and are stable in vitro for up to 21 days when cultured in human CSF [29, 41] (Fig. 5A-C). αS pff were applied on top of each culture in the same concentration as previously used for the mouse cultures (1 μl, 35 μM). In contrast to the murine cultures, however, αS pff were already applied after 3 days in culture (compared to 10 days for the murine culture) because of the overall shorter culturing time-span for human cultures. To mimic the tg mouse cultures, hA53T-αS was also expressed in the human cultures (at day 1) using an adeno-associated virus (AAV-hA53T-αS).

Consistent with the murine cultures, 1 week after the seed application, pS129-positive inclusions were apparent in AAV-hA53T-αS-treated human cultures (Fig. 5D, E). In contrast, lesions were absent in cultures treated with either αS pff or AAV-hA53T-αS alone, or in untreated controls (Fig. 5F). The pS129-positive inclusions were neuronal and also positive for pFTAA. No pFTAA-positive microglial inclusions were observed at this time point. Similar to observations in murine cultures, the induced αS inclusions in these human cultures appeared as smooth, fibrillar structures in the cytoplasm with pathology also extending into the processes (Fig. 5D, E).

## Discussion

Neurodegenerative diseases are characterised by proteopathic lesions which develop many years, if not decades before the first symptoms and lesions at death are likely to be different from the ones driving the disease [16]. Therefore, understanding the critical early phase of seeding and finding biomarkers to monitor disease progression require disease relevant models. Here we present αS lesion development in a mouse hippocampal slice culture model and find that microglial inclusions and NfL release follow neuronal αS inclusions. Moreover, we show that NfL release and spreading of αS lesions can be inhibited by a human αS antibody. Finally, we demonstrate that αS lesions can also be induced in adult human brain slice cultures.

Previous efforts to develop organotypic murine slice cultures of synucleinopathies mainly used viral overexpression of αS [42]. In this study, we expand these endeavours and show that one-time seeding of brain-derived or synthetic αS seeds is sufficient for the induction and subsequent spreading of αS lesions in murine hippocampal slice cultures. Neuronal αS inclusions in the cultures appear mature and their biochemical properties (phosphorylation of serine 129 and sarkosyl insolubility) similar to previously observed in cultures [43, 44], as wells as αS-transgenic or wildtype mice in which the lesions have been induced by seeded aggregation [9–12]. The present findings of slower αS lesions induction and overall lower burden of pathology in wt compared to tg cultures are in line with observations in mouse models [9, 10] and can be linked to enhanced vulnerability of neuronal populations with high αS expression levels [45, 46]. The morphology of the induced neuronal αS lesions in the cultures was dependent on the αS seed type (αS-tg mouse brain-derived or synthetic αS pff), again, similar to what has been described for seeded induction in mice [9–11, 47–49]. These observations are consistent with prion-like templated propagation [4] and structural differences between pff and brain-derived human αS seeds [50]. In the slice cultures as well as in mice, highly concentrated αS pff are more seeding active compared to the tg mouse brain material. Hence, when comparing the specific activity of αS pff with tg brain-derived aggregates (seed activity per αS molecules), brain-derived αS seeds appear to be more potent in seeding aggregation consistent with other proteopathic seeds [16].

The finding that αS lesions in murine slice cultures are positive for the amyloid-binding dye pFTAA is in line with recent data in humans using pFTAA and other luminescent-conjugated oligothiophenes [51, 52]. This not only confirms the amyloid nature of the inclusions in the cultures, but it also opens up the possibility to use these fluorescent dyes for live imaging of αS lesions and for spectral discrimination of αS conformers [52–54].

A growing body of research has pinpointed the importance of neuroinflammation as an essential contributor to the pathogenesis of synucleinopathies [55]. As recently reported for αS tg mice [36], we also found in the cultures pFTAA- and ThioS-positive (but largely pS129-negative) inclusions in microglia. However, microglial inclusions developed with a delay of 2–3 weeks compared to the neuronal inclusions. The local appearance of the microglia inclusions was always linked to the neuronal inclusions both in magnitude and location. Intriguingly, targeting αS seeds with an αS antibody blocked both, neuronal and microglial inclusions. Although final proof of the nature and conformation of the microglia inclusions remains to be established, the present observations are consistent with the view that the microglial inclusions contain αS of neuronal origins [36] and that microglia are somehow involved in the spreading of αS lesions in brain [56].

Modelling proteopathic lesions in slices with a brain-like environment bears the advantage that the cell-to-cell transfer of αS aggregates between interconnected regions may occur in a similar way to that in vivo [16, 57]. Immunotherapy targeting aggregated αS is a vigorously pursued therapeutic strategy, although its mechanism of action remains to be further investigated. Since it is not expected that antibodies readily enter intact neurons, αS antibodies that neutralise αS seeds are thought to capture the seeds extracellularly when transferred from cell to cell [58–62]. The observation that an αS antibody was capable of reducing the number of αS inclusions in DG and CA1 where the αS lesions spread, but less so in the CA3 injection area, suggests that this human antibody can inhibit the spreading of αS seeds. Although, the current results do not provide a conclusive explanation for the mechanisms involved, our results demonstrate the utility of this culture system for studying αS-targeting disease-modifying drugs.

Early biomarkers that indicate the initial development of proteopathic lesions before the occurrence of clinical symptoms are essential for early diagnosis and disease progression monitoring. An increase of NfL in CSF and blood has been observed in αS-tg mice as well as in human synucleinopathies [38, 63]. The present findings that αS lesions and associated neurodegeneration in murine slice cultures can be monitored by measuring NfL levels in the culture medium reveals the culture system as an important tool for translational research. NfL measurements in cultures provide even some advantages over animal models (i.e. NfL in CSF) since longitudinal measurements are possible and changes of NfL levels can be directly related to lesions as well as to therapeutic efforts.

While murine slice cultures now allow to study the formation, spreading, and targeting of the inclusions in a brain-like environment, it is important to acknowledge their limitations given that these cultures are derived from postnatal brain and therefore lack the aspect of aging. Furthermore, recent transcriptome studies revealed differences in disease-relevant genes between mouse and human cell populations [64–68]. Thus, the here described proof-of-principle translation of the slice culture model from mouse to human appears a first step forward and will allow to confirm findings derived from mouse cultures in a true aged human brain environment.

Human long-term slice cultures have only recently been developed [29, 69]. The cultures used in the present study were derived from three individuals (22–61 years old) of both sexes. Since this small sample size does not allow any conclusions about the effects of age and gender, such parameters could be addressed in the future by increasing the donor sample size. In particular, it will be interesting to study whether αS lesion induction is more prominent in aged donors compared to younger donors. Other open questions are whether longer culturing of the human cultures will allow the induction of αS lesions without additional overexpression of A53T-αS and whether microglia inclusions will also develop at prolonged culturing.

The induction of αS lesions in adult human brain cultures is an exciting step forward. However, limitations remain and need to be overcome before the human culture model can be used in a routine lab environment. The availability of suited resection tissue is limited and long-term cultivation of human cultures appears more difficult compared to the murine cultures. In addition, human CSF is needed to nurture the human cultures whose availability is also limited. Thus, at present, the human slice culture model is best used to confirm findings from the mouse cultures in a true aged human brain environment.

## Conclusion

We have established a quantitative cellular model system for studying α-synucleinopathy that contains essential elements of in vivo tissue complexity and replicates key aspects of disease. We further provided proof-of-principle evidence for their clinical application in screening antibodies that prevent the spread of αS lesions. As was the case in animal models and in humans, NfL measurements can be used as reliable readouts of disease in these slice cultures. Given the limitations of studying human disease in the context of an animal brain, we successfully translated this model from mouse to human and report the first induction of human αS lesions in a true adult human brain environment. Although our study was focussed on the induction of α-synucleinopathies, these slice culture models can be expanded to other proteopathies. Slice cultures allow for easy access and manipulation of the tissue while keeping the 3Rs (reduce, refine, replace) guiding principles. In particular, live-imaging of the pathology development with fluorescent amyloid dyes combined with single cell transcriptomics offer great potential to study human neurodegenerative diseases in new light.

## Supplementary Information

**Additional file 1: SupplementaryFig. 1**. αS pff characterization. (A, B) Proteolytic profiles of monomeric (A) and fibrillar (B) αS (100 M in PBS) after proteinase K treatment (3.7 μg/μl) at 37 °C. Aliquots were removed from the reaction at the time indicated (in min), immediately denatured with Laemmli buffer at 90 °C for 5 min and analysed on 15% PAGE. The gels were stained with Coomassie blue. The molecular weight markers are shown on the left of each gel. The αS fibrils reveal the typical proteolytic pattern as reported previously [30, 31]. (C, D) Transmission electron micrographs of the αS fibrils before (C) and after fragmentation (D). The scale bar represents 200 nm.

**Additional file 2: Supplementary Fig. 2** Induction of αS aggregation is dependent on endogenous αS expression. Immunofluorescence staining for pS129 showed inclusions in Thy1-h[A53T]αS tg HSCs seeded with Thy1-h[A53T]αS tg brain homogenate. In contrast, there was no induction of αS aggregation in Snca−/− HSCs after seeding using the same brain homogenate as seed. Analysis was done 5 weeks after treatment. See Fig. 1 for methodological detail. The experiment was done twice with 3 cultures each. Scale bar = 500 μm.

**Additional file 3: Supplementary Fig. 3** αS aggregates in αS pff and tg brain homogenate. (A, B) HTRF-FRET immunoassay analysis of aggregated αS in αS pff (light grey), tg brain homogenate (dark grey), and wt homogenate (white) at different sample dilutions. Data is reported as ratio of 665 nm / 620 nm × 10,000. Note that αS pff showed Hook effect at dilutions < 1: 51200 (Hook effect: high aggregate concentrations will capture all antibodies leading to a plateau and to a decrease of the signal) (A). Dotted line (red) illustrates exemplary comparison of dilutions that resulted in similar aggregation signal (B). Aggregation levels that gave a signal ratio of ~ 20,000 for ratio of 665 nm / 620 nm × 10,000. αS pff (light grey) needed to be diluted approximately 204,800-fold, and tg brain homogenate (dark grey) 25-fold. Although the signal cannot be attributed to an absolute amount of aggregated αS given that the conformations of aggregated αS in pff and tg brain extract are likely to be different, results appear consistent with tg brain-derived αS seeds to be more seeding potent compared to αS pff seeds. Mean ± SEM; *n* = 3 triplicate measurements per dilution.

**Additional file 4: Supplementary Fig. 4**. Time course of seeded αS aggregation in Thy1-h[A53T]αS tg mice. (A, B) Immunohistochemistry for pS129 (black) of DG from Thy1-h[A53T]αS tg mice injected at 3–4 months of age with Thy1-h[A53T]αS tg brain homogenate (A) or wt brain homogenate (B) at one day post-injection (dpi), 7 dpi or 30 dpi. For each group, three mice were used. Sections were counter stained using nuclear fast red. Note that the same brain homogenates were used for slice culture experiments. (A) First inclusions appeared around 7 dpi in all three mice and became more abundant by 30 dpi. (B) In contrast, no inclusions were found in wt brain homogenate-treated mice at any time point analysed. Scale bar represents 100 μm.

**Additional file 5: Supplementary Fig. 5**. Treatment with highly concentrated αS pff is neurotoxic to Thy1-h[A53T]αS tg HSCs. (A) Immunostaining of pS129-positive aggregates in 50 μm-horizontal sections from Thy1-h[A53T]αS tg HSCs treated with 350 μM αS pff at 1, 2 and 5 weeks post-seeding. Scale bar = 500 μm. (B) Measurement of culture thickness (in μm at the time of fixation) in untreated, 350 μM αS pff-treated and 35 μM αS pff-treated tg HSCs. Mean ± SEM; n(untr., 1 week) = 6, n(untr., 3 weeks) = 18, n(untr., 5 weeks) = 19, n(35 μM pff, 1 week) = 15, n(35 μM pff, 2 weeks) = 10, n(35 μM pff, 3 weeks) = 31, n(35 μM pff, 4 weeks) = 9, n(35 μM pff, 5 weeks) = 18, n(350 μM pff, 1–4 weeks) = 6 each, n(350 μM pff, 5 weeks) = 35 HSCs per group. Note that culture thickness was routinely measured and results come from many different experiments, which accounts for the differences among the n/group. **(C)** Violin graphs displaying thickness of tg HSCs across different treatments at 5 weeks post-treatment. n(tg, untr.) = 19, n(tg, tg brain hom.) = 24, n(tg, 35 μM pff) = 18, n(tg, 350 μM pff) = 35. Kruskal-Wallis test (H = 45.82; *p* < 0.0001): **p < 0.0001 Dunn’s multiple comparisons against untreated HSCs. **(D)** Violin graphs displaying thickness of wt HSCs across different treatments at 5 weeks post-treatment. n(wt, untr.) = 21, n(wt, tg brain hom.) = 27, n(wt, 35 μM pff) = 3, n(wt, 350 μM pff) = 18. Kruskal-Wallis test (H = 11.64; *p* = 0.0087): **p < 0.01 Dunn’s multiple comparisons against untreated HSCs.

## Data Availability

The datasets are available from the corresponding author upon reasonable request.

## Abbreviations

Aβ: Amyloid-beta
αS: Alpha-synuclein
AAV: Adeno-associated virus
CA: Cornu ammonis
CSF: Cerebrospinal fluid
DG: Dentate gyrus
DLB: Dementia with Lewy bodies
HSC: Hippocampal slice culture
MSA: Multiple system atrophy
NfL: Neurofilament light chain
PD: Parkinson’s disease
PFA: Paraformaldehyde
PFF: Preformed fibrils
TG: Transgenic
WB: Western blot
WT: Wild type
