# Supplementary figures and images for: Microglial inclusions and neurofilament light chain release follow neuronal α-synuclein lesions in long-term brain slice cultures
Source: Mol Neurodegener. 2021 Aug 11;16:54. doi: 10.1186/s13024-021-00471-2 (PMC8356412; doi:10.1186/s13024-021-00471-2)

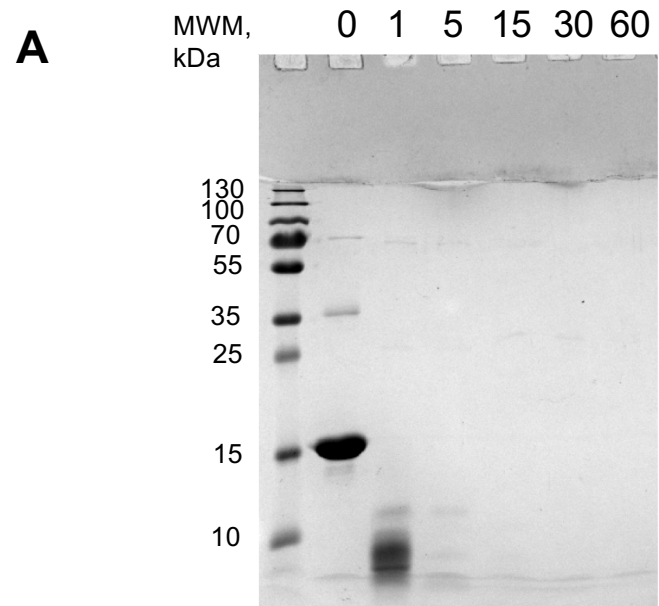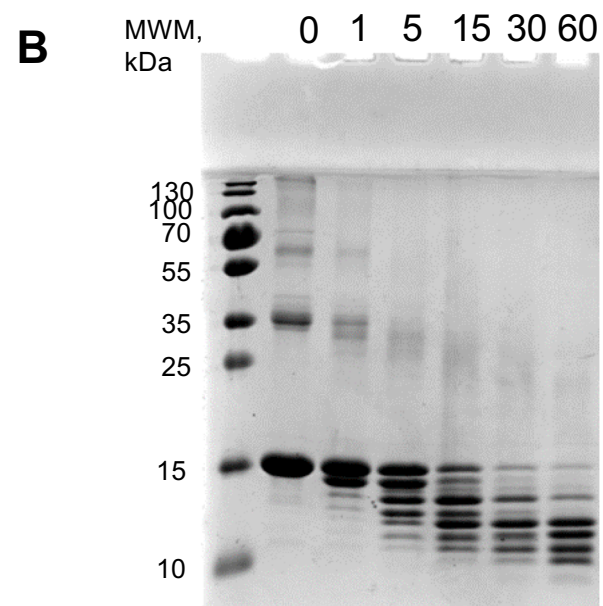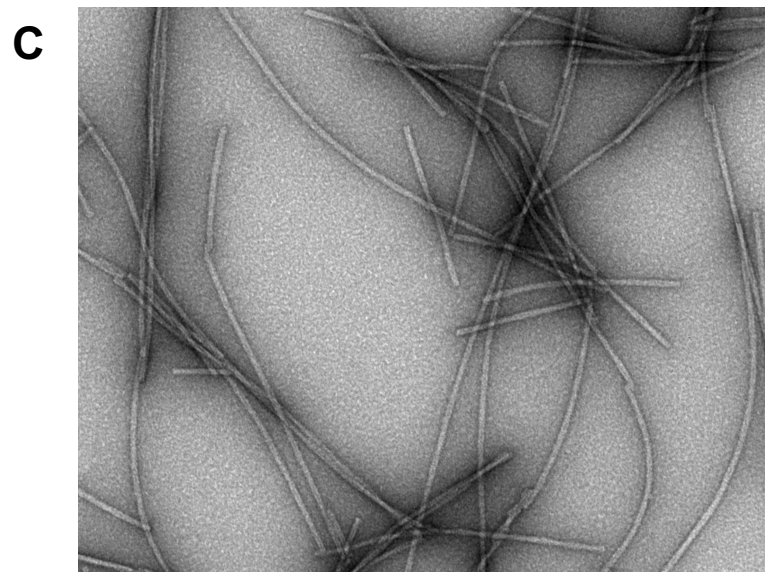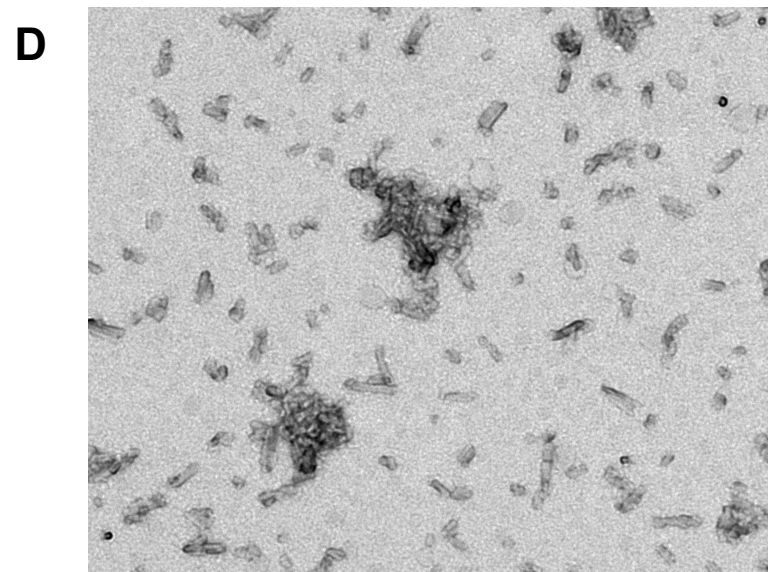

Supplement: Supplementary file 1 — Additional file 1: SupplementaryFig. 1. αS pff characterization. (A, B) Proteolytic profiles of monomeric (A) and fibrillar (B) αS (100 M in PBS) after proteinase K treatment (3.7 μg/μl) at 37 °C. Aliquots were removed from the reaction at the time indicated (in min), immediately denatured with Laemmli buffer at 90 °C for 5 min and analysed on 15% PAGE. The gels were stained with Coomassie blue. The molecular weight markers are shown on the left of each gel. The αS fibrils reveal the typical proteolytic pattern as reported previously [30, 31]. (C, D) Transmission electron micrographs of the αS fibrils before (C) and after fragmentation (D). The scale bar represents 200 nm. [file 13024_2021_471_MOESM1_ESM.pdf]

Tg host

Ko host

+ tg brain extract

5 weeks

pS129

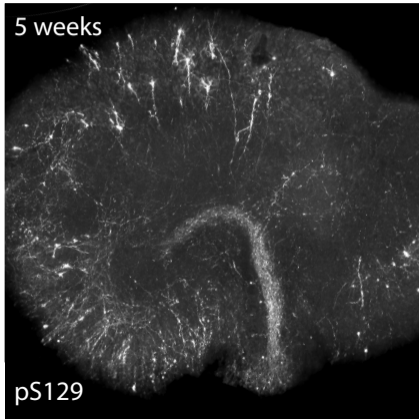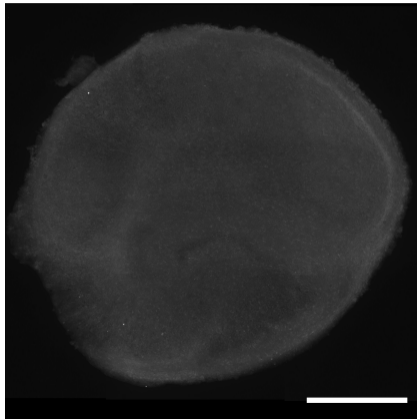

Supplement: Supplementary file 2 — Additional file 2: Supplementary Fig. 2 Induction of αS aggregation is dependent on endogenous αS expression. Immunofluorescence staining for pS129 showed inclusions in Thy1-h[A53T]αS tg HSCs seeded with Thy1-h[A53T]αS tg brain homogenate. In contrast, there was no induction of αS aggregation in Snca−/− HSCs after seeding using the same brain homogenate as seed. Analysis was done 5 weeks after treatment. See Fig. 1 for methodological detail. The experiment was done twice with 3 cultures each. Scale bar = 500 μm. [file 13024_2021_471_MOESM2_ESM.pdf]

**A**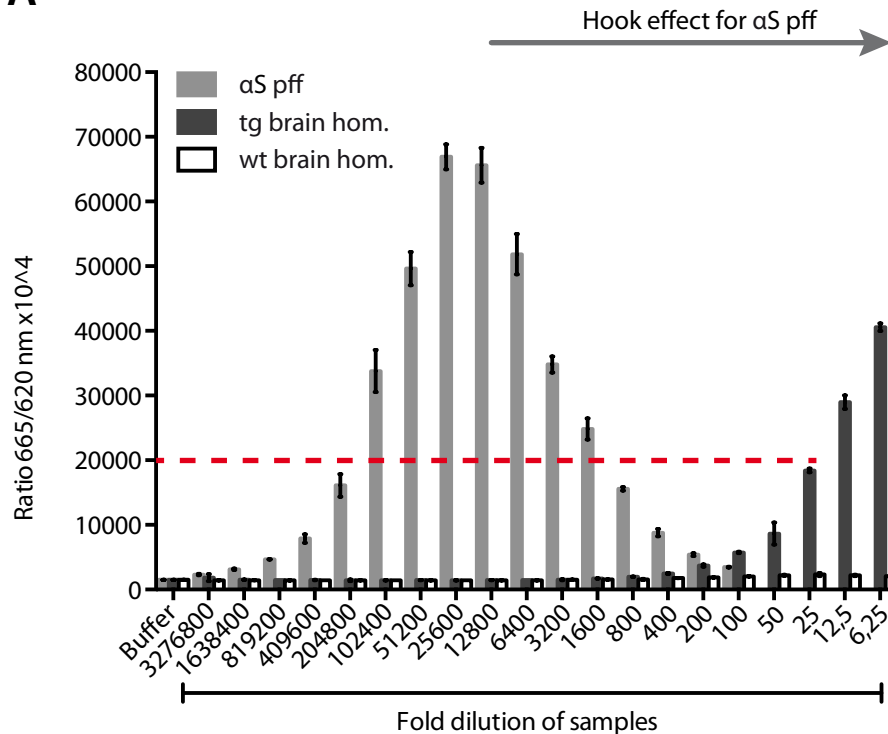**B**

dilution comparison  
for signal ratio  $\sim 20\,000$

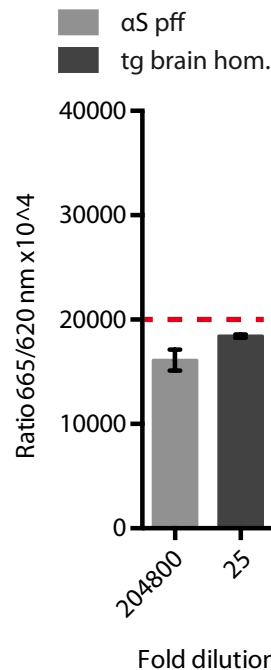

Supplement: Supplementary file 3 — Additional file 3: Supplementary Fig. 3 αS aggregates in αS pff and tg brain homogenate. (A, B) HTRF-FRET immunoassay analysis of aggregated αS in αS pff (light grey), tg brain homogenate (dark grey), and wt homogenate (white) at different sample dilutions. Data is reported as ratio of 665 nm / 620 nm × 10,000. Note that αS pff showed Hook effect at dilutions < 1: 51200 (Hook effect: high aggregate concentrations will capture all antibodies leading to a plateau and to a decrease of the signal) (A). Dotted line (red) illustrates exemplary comparison of dilutions that resulted in similar aggregation signal (B). Aggregation levels that gave a signal ratio of ~ 20,000 for ratio of 665 nm / 620 nm × 10,000. αS pff (light grey) needed to be diluted approximately 204,800-fold, and tg brain homogenate (dark grey) 25-fold. Although the signal cannot be attributed to an absolute amount of aggregated αS given that the conformations of aggregated αS in pff and tg brain extract are likely to be different, results appear consistent with tg brain-derived αS seeds to be more seeding potent compared to αS pff seeds. Mean ± SEM; n = 3 triplicate measurements per dilution. [file 13024_2021_471_MOESM3_ESM.pdf]

**A**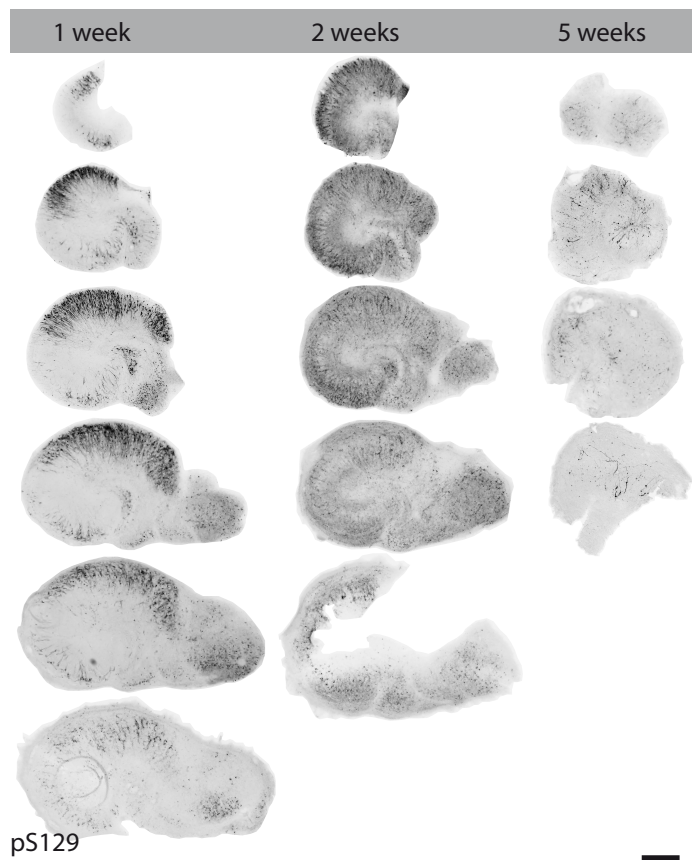**B**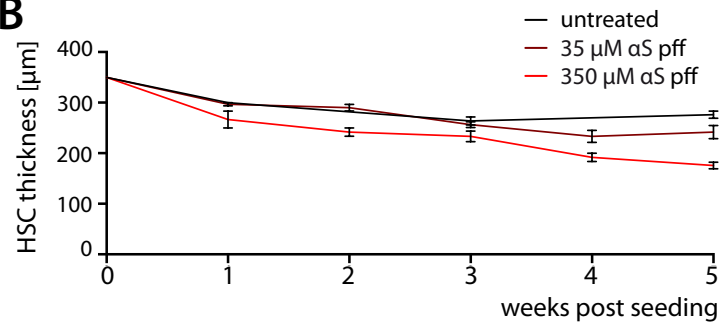**C**

tg HSC, 5 weeks

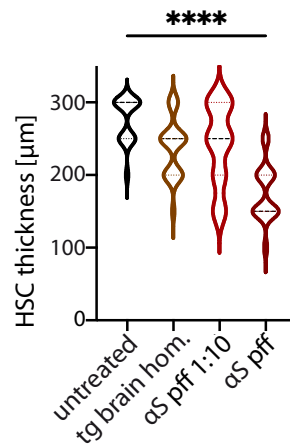**D**

wt HSC, 5 weeks

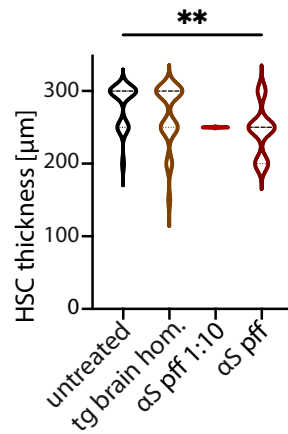

Supplement: Supplementary file 5 — Additional file 5: Supplementary Fig. 5. Treatment with highly concentrated αS pff is neurotoxic to Thy1-h[A53T]αS tg HSCs. (A) Immunostaining of pS129-positive aggregates in 50 μm-horizontal sections from Thy1-h[A53T]αS tg HSCs treated with 350 μM αS pff at 1, 2 and 5 weeks post-seeding. Scale bar = 500 μm. (B) Measurement of culture thickness (in μm at the time of fixation) in untreated, 350 μM αS pff-treated and 35 μM αS pff-treated tg HSCs. Mean ± SEM; n(untr., 1 week) = 6, n(untr., 3 weeks) = 18, n(untr., 5 weeks) = 19, n(35 μM pff, 1 week) = 15, n(35 μM pff, 2 weeks) = 10, n(35 μM pff, 3 weeks) = 31, n(35 μM pff, 4 weeks) = 9, n(35 μM pff, 5 weeks) = 18, n(350 μM pff, 1–4 weeks) = 6 each, n(350 μM pff, 5 weeks) = 35 HSCs per group. Note that culture thickness was routinely measured and results come from many different experiments, which accounts for the differences among the n/group. (C) Violin graphs displaying thickness of tg HSCs across different treatments at 5 weeks post-treatment. n(tg, untr.) = 19, n(tg, tg brain hom.) = 24, n(tg, 35 μM pff) = 18, n(tg, 350 μM pff) = 35. Kruskal-Wallis test (H = 45.82; p < 0.0001): **p < 0.0001 Dunn’s multiple comparisons against untreated HSCs. (D) Violin graphs displaying thickness of wt HSCs across different treatments at 5 weeks post-treatment. n(wt, untr.) = 21, n(wt, tg brain hom.) = 27, n(wt, 35 μM pff) = 3, n(wt, 350 μM pff) = 18. Kruskal-Wallis test (H = 11.64; p = 0.0087): **p < 0.01 Dunn’s multiple comparisons against untreated HSCs. [file 13024_2021_471_MOESM5_ESM.pdf]
