# Supplementary material for: Microglial inclusions and neurofilament light chain release follow neuronal α-synuclein lesions in long-term brain slice cultures
Source: Mol Neurodegener. 2021 Aug 11;16:54. doi: 10.1186/s13024-021-00471-2 (PMC8356412; doi:10.1186/s13024-021-00471-2)
Supplement: Supplementary file 4 — Additional file 4: Supplementary Fig. 4. Time course of seeded αS aggregation in Thy1-h[A53T]αS tg mice. (A, B) Immunohistochemistry for pS129 (black) of DG from Thy1-h[A53T]αS tg mice injected at 3–4 months of age with Thy1-h[A53T]αS tg brain homogenate (A) or wt brain homogenate (B) at one day post-injection (dpi), 7 dpi or 30 dpi. For each group, three mice were used. Sections were counter stained using nuclear fast red. Note that the same brain homogenates were used for slice culture experiments. (A) First inclusions appeared around 7 dpi in all three mice and became more abundant by 30 dpi. (B) In contrast, no inclusions were found in wt brain homogenate-treated mice at any time point analysed. Scale bar represents 100 μm. [file 13024_2021_471_MOESM4_ESM.pdf]

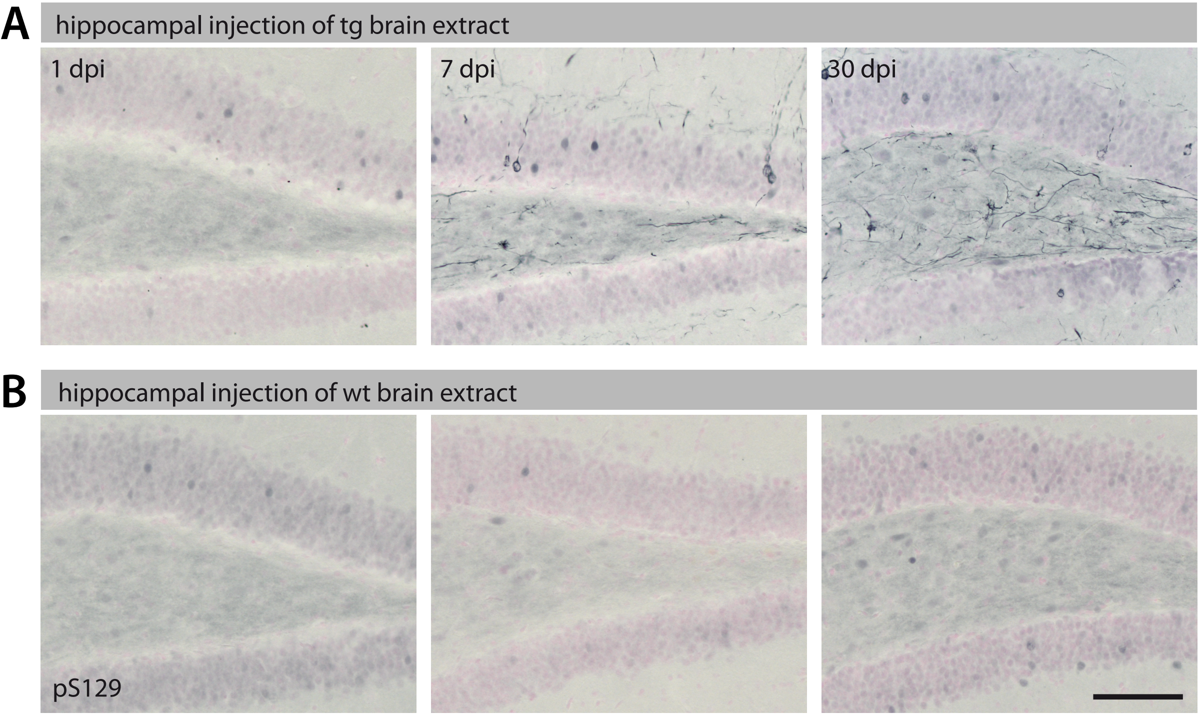
A
hippocampal injection of tg brain extract
1 dpi
7 dpi
30 dpi
B
hippocampal injection of wt brain extract
pS129
